# The Role of the Innate Immune System in ALS

**DOI:** 10.3389/fphar.2012.00150

**Published:** 2012-08-14

**Authors:** Sudarshan Phani, Diane Berengere Re, Serge Przedborski

**Affiliations:** ^1^Department of Pathology and Cell Biology, Columbia UniversityNew York, NY, USA; ^2^Center for Motor Neuron Disease, Department of Neurology, Columbia UniversityNew York, NY, USA

**Keywords:** amyotrophic lateral sclerosis, astrocyte, microglia, motor neuron, innate immune system

## Abstract

Amyotrophic lateral sclerosis (ALS) is a fatal, adult-onset neurodegenerative disease that is characterized by the death of upper and lower motor neurons. Recent studies have made it clear that although motor neurons are the primary targets of the degenerative process, other cell types play key roles in the death of motor neurons. Most notably, cells of the immune system, including astrocytes and microglia have come under increasing scrutiny, after multiple lines of evidence have shown these cells to be deleterious to motor neurons. Both *in vitro* and *in vivo* experiments have shown that astrocytes and microglia containing mutated SOD1 are harmful to motor neurons. Several studies on ALS and other neurodegenerative diseases have revealed that reactive astrocytes and microglia are capable of releasing pro-inflammatory factors such as cytokines and chemokines, which are harmful to neighboring neurons. In addition, it is believed that diseased astrocytes can specifically kill motor neurons through the release of toxic factors. Furthermore, in an animal model of the disease, it has been shown that the reduction of SOD1 in microglia may be able to slow the progression of ALS symptoms. Although the exact pathways of motor neuron death in ALS have yet to be elucidated, studies have suggested that they die through aBax-dependent signaling pathway. Mounting evidence suggests that neuroinflammation plays an important role in the degeneration of motor neurons. Based on these findings, anti-inflammatory compounds are currently being tested for their potential to reduce disease severity; however, these studies are only in the preliminary stages. While we understand that astrocytes and microglia play a role in the death of motor neurons in ALS, much work needs to be done to fully understand ALS pathology and the role the immune system plays in disease onset and progression.

## Introduction

Amyotrophic Lateral Sclerosis (ALS) is a fatal adult-onset neurodegenerative disorder that is characterized clinically by muscle weakness and wasting. Pathologically, it is identified by the degeneration of upper and lower motor neurons (Boillee et al., 2006a). Generally, death results from respiratory failure due to paralysis of the respiratory muscles. The incidence of ALS is reported to be between 1.5 and 3 per 100,000 in Europe and North America (Wijesekera and Leigh, 2009; Hardiman et al., 2011). ALS onset peaks between the ages of 50–75 and declines thereafter. These factors suggest that ALS may not be a disease of aging, but rather that age may be one of a multitude of contributing factors (Hardiman et al., 2011). Thus far, there are no effective treatments and no cure for ALS.

Amyotrophic lateral sclerosis is essentially a sporadic disorder, with greater than 90% of the cases originating from an unknown cause. However, approximately 10% of the cases are considered familial (FALS) and are generally inherited as a dominant disorder (Ince et al., 2011) due to mutations in a number of seemingly disparate genes. These include, but are not limited to, mutations in genes such as TAR DNA binding protein (TDP)-43 and Fused in Sarcoma (FUS) (Sreedharan et al., 2008; Kwiatkowski et al., 2009; Vance et al., 2009). Despite major enthusiasm by the research community about the recent discoveries of mutations in FUS and TDP-43, the best characterized form of FALS still remains that which is linked to mutations in the copper-zinc superoxide dismutase gene (SOD1) and which accounts for 10–20% of all FALS cases (Andersen, 2001). Myriad mutations in SOD1 all seem to give rise to an ALS phenotype via a toxic gain-of-function mechanism, which is clinically almost indistinguishable from sporadic cases. Histopathologically, however, recent studies have found TDP-43 to be a reliable marker for differentiating sALS from SOD1-related fALS. These studies have shown that all of the cases of sporadic ALS and SOD1-negative fALS had neural and glial inclusions which were immunoreactive for both ubiquitin and TDP-43, whereas fALS cases with mutations in SOD1 were universally absent of TDP-43 immunoreactivity (MacKenzie et al., 2007; Tan et al., 2007).

More recently, studies have identified an expanded GGGGCC hexanucleotide repeat in a non-coding region of C9ORF72. This expansion has been shown to be linked to chromosome 9p-linked FTD and ALS. Interestingly, this same repeat expansion was also identified in the majority of families with a combined FTD/ALS phenotype and TDP-43 pathology. Extended studies have identified the C9ORF72 expansion as the most common genetic abnormality in fALS (23.5%; DeJesus-Hernandez et al., 2011).

A number of studies have pointed to oxidative stress, protein aggregation, or mitochondrial dysfunction as mechanisms of mutant SOD1 toxicity. In addition, SOD1 toxicity has been shown to be linked to changes in the immune system, which has also been shown to contribute to the motor neuron pathology seen in ALS; however, the exact nature of the pathogenic mechanisms provoking motor neuron degeneration in mutant SOD1-linked ALS remains elusive (Ince et al., 2011). Interestingly, the repeat expansion in C9ORF72 results in the formation of nuclear RNA foci not seen in SOD1-linked pathology, suggesting multiple disease mechanisms (DeJesus-Hernandez et al., 2011).

While the discovery of the C9ORF72 repeat expansion has opened new avenues of ALS research, the abundance and well-studied nature of SOD1-mutant animals make them the *de facto* models of choice to study ALS pathology for the time being. Although imperfect, these models allow for a great deal of insight into potential mechanisms involved in ALS pathology, with the hope that the mechanisms elucidated through the use of these models may also provide understanding of sALS and non-SOD1-mediated fALS.

## Inflammation and Neurodegeneration

Although ALS is a disease primarily affecting upper and lower motor neurons, it is increasingly recognized that the entire pathogenic process of ALS is not restricted to a set of cell-autonomous deleterious mechanisms taking place within motor neurons. Instead, it is now believed that non-cell autonomous mechanisms, such as neuroinflammation may also contribute to the disease process.

Germane to this issue is the fact that the immune system has been found to be altered in sporadic ALS. Studies have shown immunological differences in the blood of ALS patients compared to healthy controls. These include increased levels of CD4+ cells, and reduced CD8+ T-lymphocytes (Mantovani et al., 2009). Interestingly, blood samples analyzed from patients at an earlier and less severe stage of the disease also show altered expression of immune cells, such as significant reductions in CD4+CD25+ T-regulatory (T-reg) cells as well as CD14+ monocytes (Mantovani et al., 2009). Additionally, T-reg cells have been shown to play significant roles as neuroprotectants responsible for modulating the neuroinflammatory response in mouse models of neurodegeneration (Kipnis et al., 2004). It is therefore possible to hypothesize that the reduction of T-reg cells in the blood of sporadic ALS patients might represent a recruitment of these cells from the periphery into the CNS in order to activate resident innate immune cells such as microglia, as well as anti-inflammatory cytokines such as interleukin-10 and transforming growth factor-β in an effort to protect the area most affected by the early effects of ALS degeneration (Kipnis et al., 2004; Mantovani et al., 2009).

Markers for resident innate immune cells have also been found to be altered in the brains of ALS patients as well as in animal models of ALS. For instance, immunostaining for glial fibrillary acid protein (GFAP), a common marker for astrocytes, is markedly increased in all forms of ALS in the precentral gyrus of human samples (Kawamata et al., 1992). In addition, staining for leukocyte common antigen (LCA), lymphocyte function associate molecule-1 (LFA-1), and complement receptors CR3 (CD11b) and CR4 (CD11c) are increased, supporting the idea that microglia and macrophages are activated in the areas of ALS degeneration, such as the motor cortex, brainstem, and corticospinal tract (Kawamata et al., 1992; Papadimitriou et al., 2010). Remarkably, it is believed that the early site of pathological changes in ALS is the neuromuscular junction, and while this particular site of the lower motor neuron pathway has been extensively studied, very little information exists about the immune response at that level. Nonetheless, the data reviewed above provide compelling evidence that a robust neuroinflammatory response is part of the neuropathological changes that characterize ALS. However, none of the aforementioned studies address the actual role, if any, of neuroinflammation in ALS. Investigations in other neurodegenerative disorders have shown that neuroinflammation may exert dual effects, that it can be protective or harmful. In order to distinguish between these two possibilities, efforts have generally concentrated on studying animal models of ALS.

The blood brain barrier renders the brain as immune-privileged, therefore, the brain contains resident astrocytes and microglia that perform the duties of immune surveillance and act as an innate response system. Reactive astrocytes are most commonly recruited to the site of an injury, such as a spinal cord injury or ischemic events. Their main functions are to compact inflammatory cells, and to re-establish the blood brain barrier (Okada et al., 2006) they may also be involved in clearing CNS debris, such as amyloid plaques (Papadimitriou et al., 2010). Aside from astrocytes, the CNS immune system also consists of ramified resting microglial cells, whose processes play an active role in maintaining the homeostasis of the neural environment. Once activated by the presence of antigens and during times of distress, such as is the case during neurodegeneration, microglia, like other immune cells, become activated taking on an ameboid morphology. Once activated, microglia can secrete a myriad of neurotrophic factors and cytokines (Napoli and Neumann, 2009), and engage in the clearance of pathogens and debris through phagocytosis. However, unregulated activation of microglial activation may be harmful to neurons through the release of potentially harmful neurotoxins including quinolinic acid, reactive oxygen and nitrogen species (ROS; RNS), and pro-inflammatory cytokines. Although a large number of studies have shown that microglia and, to a lesser extent, astrocytes are activated in the affected areas of the CNS in ALS, thus far little attention has been paid to the determination of the actual phenotype of these cells. For instance, since activated resident immune cells can display either a pro-inflammatory or anti-inflammatory phenotype, sometimes referred to as M1 or M2, it will be critical to determine which phenotype they exhibit as a whole, and if possible as individual cells. It is also important to determine whether such profiles differ among the various affected structures of the CNS and stages of the disease. Aside from these outstanding questions, it is also critical to elucidate whether these inflammatory cells play a role in the pathogenesis of ALS. The use of animal models is greatly aiding in the attempts to answer the questions raised by the findings described above.

The most commonly studied animal models of ALS are rats and mice expressing mutant SOD1 (mSOD1). Although, as indicated above, SOD 1 mutations only account for a small percentage of human cases, animal models using mSOD1 are used with the hope that the mechanisms leading the disease are shared across cases, and knowledge gained from these models can be used to understand the development of the disease in humans. Notably, evidence for increases in astrocytes and microglia have also been found in transgenic mice expressing different forms of mutant SOD1 (mSOD1), supporting human pathology studies (Fischer et al., 2004). Specifically, GFAP and CD11b staining showed evidence for significant increases in reactive astrocytes and microglia in the motor regions of transgenic mice containing mSOD1 (Fischer et al., 2004). Although both astrocytosis and microgliosis have been observed in rodent models, the timing of events seems to be unclear. Some studies using mSOD1 mice have suggested astrocytosis to be an early stage event, with microgliosis as a late stage reaction (Yang et al., 2011). However, others have suggested that microglial response occurs at a much earlier stage of the disease (Sanagi et al., 2010). Nevertheless, it does appear that microglial activation starts after motor neuron degeneration, and initially tries to protect motor neurons from degeneration (Fischer et al., 2004; Henkel et al., 2009).

### Immune cell mediated non-cell autonomy in ALS

Motor neuron death and degeneration has been implicated in causing the debilitating symptoms characteristic of ALS, however, mounting evidence indicates that motor neuron death is, at least in part, non-cell autonomous. *In vivo* studies have shown that mSOD1 expressed only in motor neurons of transgenic mice is either not sufficient to cause neurodegeneration or causes only a mild ALS-like phenotype (Jaarsma et al., 2008). Support for non-cell autonomy comes from studies showing that when mSOD1 expression was reduced in microglia and macrophages in transgenic mice (Boillee et al., 2006b; Wang et al., 2009), motor neuron degeneration was decreased. Aside from microglia, evidence has shown that astrocytes may also play a role in motor neuron death. Early studies using transgenic mice expressing mSOD1 in astrocytes suggested the need for motor neurons to be impaired for a degenerative phenotype (Gong et al., 2000), however more recent studies have concluded that astrocytes expressing mSOD1 released toxic factor(s) which were sufficient to cause motor neuron degeneration in an *in vitro* model of ALS (Nagai et al., 2007).

### The role of astrocytes in ALS non-cell autonomy

Astrocytes are one of the main cell types responsible for the clearance of potentially excitotoxic glutamate from the synaptic space, and as mentioned earlier, play a distinct role when they become activated by the immune system. Quite interestingly, glutamate handling has been reported to be modified in both sporadic and familial ALS (Boillee et al., 2006a). Studies have found that brain and spinal cord samples from ALS patients showed altered glutamate transport (Rothstein et al., 1992) resulting from changes in the glutamate transporter EAAT2 (Maragakis et al., 2004). Evidence suggests that EAAT2 is significantly decreased in the motor cortex and spinal cords of ALS patients (Fray et al., 1998), as well as in the spinal cords of mSOD1 transgenic mice (Bruijn et al., 1997) and rats (Howland et al., 2002). It should come as no coincidence then, that astrocytes were quickly targeted for their role in the ALS disease processes.

More interestingly in terms of inflammation, samples analyzed from the brains and spinal cords of ALS patients were found to contain greater numbers of activated or reactive astrocytes, a clear sign of an immune response (Sta et al., 2011). Of note, and useful for research purposes, increased reactive astrocyte expression has been found both in ALS patients and in animal models of ALS (Sta et al., 2011).

Although the main function of astrocytes in the synaptic space is the maintenance of a homeostatic environment for neurons though activities such as the clearance of neurotransmitters, reactive astrocytes gain properties that resting astrocytes do not exhibit. It has been shown that during and after injury, astrocytes have the ability to ameliorate symptoms by physically isolating the injured area through the formation of a glial scar, as well as release neurotrophins and growth factors such as IGF-1 which have been deemed beneficial to injured cells (Dong and Benveniste, 2001). Astrocytes have also been found to release NGF and induce additional sprouting both *in vitro* and *in vivo*, a key step in neuronal recovery (Chalmers et al., 1996; Wu et al., 1998).

Even though astrocytes do have the potential to be beneficial to neurons, the majority of evidence suggests that astrocytes actually contribute to neuronal degeneration during disease, with ALS being no exception. Interestingly, NGF release, which has generally been regarded as a positive attribute of reactive astrocytes, has been shown to directly lead to motor neuron apoptosis through the p75 pathway (Pehar et al., 2004). Notably, mSOD1-containing astrocytes have been found to be even more toxic to their environments than reactive astrocytes alone. Studies have found that reducing mSOD1 expressing astrocyte levels in an *in vivo* model of ALS decreased motor neuron degeneration and correspondingly increased the life span of the affected animals (Lepore et al., 2008; Barbeito et al., 2010). Although the exact mechanisms for astrocyte-mediated motor neuron toxicity is not known, several lines of evidence have pointed to the activation and release of toxic or harmful products such as pro-inflammatory cytokine oxidative stressors. These include, but are not limited to prostaglandins, leukotrienes, and RNS (Hensley et al., 2006a,b). In addition, *in vitro* models of ALS have shown that astrocytes containing mSOD1 release toxic factors that are preferentially toxic to motor neurons. Furthermore, it was noted that even wild-type motor neurons die in the presence of mSOD1-containing astrocytes. Moreover, wild-type motor neurons die when grown in media conditioned by mSOD1 containing astrocytes. These *in vitro* studies have suggested the pro-apoptotic Bax pathway as another mechanism through which astrocytes mediate motor neuron cell death (Nagai et al., 2007).

### The role of microglia in ALS

Microglial cells perform the duties of immune surveillance in the CNS, and once activated, such as is the case during neurodegeneration, these cells secrete cytokines and neurotrophins in order to help restore homeostasis to the neuronal environment (Napoli and Neumann, 2009). However, just as astrocytes may play a deleterious role in ALS, microglia have also been found to exacerbate neuronal injury. *In vivo* studies have shown that microglial cells increase their expression and release of pro-inflammatory cytokines, such as TNF-α and IL-1β in a mouse model of ALS. Additionally, activated microglia have been shown to promote the generation of reactive oxidative species, causing more harm than good to motor neurons (Henkel et al., 2009).

In the case of ALS, a number of studies have looked at mSOD1 expression in microglia, and its effect on ALS disease progression and motor neuron degeneration. Recent studies have found that diminishing mSOD1 levels in microglia in a mouse model of ALS did not alter the age of onset of paralysis, however it was able to significantly slow the disease progression (Boillee et al., 2006b). Yet, the lack of effects on onset must be regarded with caution, as one cannot exclude that the reduction in mSOD1 in microglia was only at the time when some actual motor neuron degeneration has already occurred. Indeed, the technology used in this study to reduce mSOD1 expression was driven by the microglial specific promoter CD11b which only acquires significant driving forces in response to pathological stimuli such as motor neuron degeneration. Accordingly, it would not be surprising that the disease phenotype in these mice has only been modified after the onset of motor neuron degeneration. It has also been found that mSOD1 containing microglia produce and release greater levels of pro-inflammatory cytokines and free radicals than their wild-type counterparts (Henkel et al., 2009). While the presence of mSOD1 in microglia has been shown to be deleterious, new evidence has begun to pinpoint the mechanisms that may control ALS-related microglial toxicity. Specifically, increases in the pro-inflammatory cytokines and prostaglandin E_2_ have been reported to be present in ALS patients (Papadimitriou et al., 2010). Additionally, *in vitro* data suggested that conditioned media obtained from activated microglia is sufficient to cause neuronal degeneration through the concurrent stimulation of TNF-α and NMDA receptors. These pathways are thought to up-regulate inducible nitric oxide synthase activity, thus causing oxidative stress which leads to cell death. Of note is the fact that activation of either the TNF-α or NMDA receptor alone was not found to cause cell death, and the blockade of either of these receptor pathways was found to alleviate cell death caused by microglial-conditioned media, suggesting that both of these receptors need to be activated for MN death to occur (Moisse and Strong, 2006).

Although there is compelling evidence supporting the notion that microglial activation could be sufficient to kill motor neurons in ALS, *in vivo* studies have cast doubt on this theory by showing that repopulating microglial-deficient mice with mSOD-containing microglia failed to cause motor neuron death. These studies have yet to be repeated (Boillee et al., 2006a,b). In addition, studies done in mSOD1 mice absent in TNF-α, a potent inflammatory signaling molecule, did not show any decrease in motor neuron death (Gowing et al., 2006). These data are perhaps suggestive of alternate inflammatory pathways, and may implicate inflammatory mechanisms as coincidental, but not causative.

When viewed together, mounting *in vitro* and *in vivo* evidence have given credence to the toxic effects of astrocytes and microglia on motor neurons and have suggested multiple mechanisms that may be involved. In addition to these, it must be noted that mSOD1 containing motor neurons are particularly vulnerable to Fas ligand and NO-triggered cell death (Moisse and Strong, 2006). Therefore, it is thought that the combined effects of mSOD1 in motor neurons as well as in neighboring astrocytes and microglia are what ultimately result in cell death and degeneration in ALS. While the discovery of the expansion repeat in C9ORF72 is too recent to know its relationship with the inflammatory system, it would be conceivable that the immune system response to such a widespread mutation would be equal to or greater than what has already been observed in other forms of ALS.

### Anti-inflammatory therapeutics in ALS

With inflammation potentially playing a significant role in ALS pathogenesis and progression, it is logical to attempt anti-inflammatory drugs for use as therapeutics in combating ALS. Using SOD1 mice, a number of groups have attempted to administer COX2 inhibitors such as cyclosporine, thalidomide, and lenalidomide to COX2-deficient mice in order to attenuate the negative effects of inflammation. Interestingly, the use of these drugs in mSOD1 animal models was sufficient to prolong lifespan (Karlsson et al., 2004; Kiaei et al., 2006). Cyclosporine is currently in clinical trials. Initial double-blind, placebo-controlled clinical trials suggest that men may benefit from cyclosporine administration when given within 18 months of initial diagnosis (Appel et al., 1988). Based on these results, the group of Karlsson et al. are currently running Phase II clinical trials.

Another approach to anti-inflammatory therapy was the use of the drug Copaxone, which has most often been used to treat patients diagnosed with multiple sclerosis. The active ingredient, glatiramer acetate, was used both in animal models as well as clinical trials. While there was initial excitement for the drug, which increased survival in mSOD1 mouse models of ALS (Angelov et al., 2003), these results could not be replicated (Turner and Talbot, 2008). Clinical trials with glatiramer acetate were also unsuccessful (Meininger et al., 2009), suggesting the mouse strain or the delivery mechanism in the original study may have played a greater role than initially indicated.

Perhaps the most well-known of the anti-inflammatory compounds used against ALS is minocycline, a second-generation tetracycline which has been shown to have anti-inflammatory properties (Yrjanheikki et al., 1999). The use of minocycline was shown to delay motor neuron degeneration as well as increase survival in several different mSOD1 mouse models of ALS (Kriz et al., 2002; Zhu et al., 2002). These studies found that minocycline may have had a direct impact on motor neurons by decreasing apoptosis through a reduction in cytochrome *c* release (Zhu et al., 2002; Moisse and Strong, 2006). Furthermore, it may have decreased the level of microglial activation and proliferation in mSOD1 mouse models of ALS (Kriz et al., 2002; Moisse and Strong, 2006). Given these promising results, minocycline was moved into clinical trials, and thus far, no observable difference was found between placebo and drug cohorts in phase III trials (Barbeito et al., 2010).

It should be noted however, that the use of anti-inflammatory drugs in animal models does not lead to direct knowledge of drug dosing and further study may be needed in order to find correct dosing levels, and potentially more importantly, dosing schedules. The use of animal models allows researchers to administer therapeutics before, during, after, or any combination therein relative to symptom onset. Unfortunately, patients being treated for neurodegenerative disorders do not have the luxury of being pre-treated for ALS symptoms before they arise, thus vastly complicating any therapeutic regimen.

## Conclusion

Recent lines of evidence have given strong support to the role of astrocytes and microglia in ALS disease pathology. Tissue samples from ALS patients have been found to contain increased inflammatory by-products, and animal models of ALS have corroborated these increases. There is some debate as to whether inflammation in ALS could potentially be protective, however most studies have found the activation of microglia and astrocytes to be more harmful than beneficial. These harmful effects may come through the up-regulation and increased release of pro-inflammatory cytokines and reactive oxidative species, as well as through decreased uptake of excitotoxic neurotransmitters such as glutamate from the synaptic space. One major unanswered question, however, is whether an external event causes neuroinflammatory activation leading to ALS, or whether an external event leads to ALS causing neuroinflammation. In either case, both ALS pathology and neuroinflammation seem to act concurrently to exacerbate the symptoms of neurodegeneration. Future therapies to combat motor neuron death may involve a combination of approaches which recognize the role of multiple mechanisms and cell types in the progression of ALS.

## Conflict of Interest Statement

The authors declare that the research was conducted in the absence of any commercial or financial relationships that could be construed as a potential conflict of interest.
